# Bioclimatic atlas of the terrestrial Arctic

**DOI:** 10.1038/s41597-023-01959-w

**Published:** 2023-01-19

**Authors:** Mika Rantanen, Matti Kämäräinen, Pekka Niittynen, Gareth K. Phoenix, Jonathan Lenoir, Ilya Maclean, Miska Luoto, Juha Aalto

**Affiliations:** 1 Finnish Meteorological Institute, Helsinki, Finland; 2 Department of Biological and Environmental Science, University of Jyväskylä, Jyväskylä, Finland; 3 Department of Geosciences and Geography, University of Helsinki, Helsinki, Finland; 4 Plants Photosynthesis and Soil, School of Biosciences, University of Sheffield, Sheffield, S10 2TN United Kingdom; 5 UMR CNRS 7058, Ecologie et Dynamique des Systèmes Anthropisés (EDYSAN), Université de Picardie Jules Verne, Amiens, France; 6 Environment & Sustainability Institute, University of Exeter Penryn Campus, Penryn, TR10 9FE United Kingdom

**Keywords:** Biogeography, Climate-change ecology, Climate change

## Abstract

The Arctic is the region on Earth that is warming at the fastest rate. In addition to rising means of temperature-related variables, Arctic ecosystems are affected by increasingly frequent extreme weather events causing disturbance to Arctic ecosystems. Here, we introduce a new dataset of bioclimatic indices relevant for investigating the changes of Arctic terrestrial ecosystems. The dataset, called ARCLIM, consists of several climate and event-type indices for the northern high-latitude land areas > 45°N. The indices are calculated from the hourly ERA5-Land reanalysis data for 1950–2021 in a spatial grid of 0.1 degree (~9 km) resolution. The indices are provided in three subsets: (1) the annual values during 1950–2021; (2) the average conditions for the 1991–2020 climatology; and (3) temporal trends over 1951–2021. The 72-year time series of various climate and event-type indices draws a comprehensive picture of the occurrence and recurrence of extreme weather events and climate variability of the changing Arctic bioclimate.

## Background & Summary

Over the last four decades, the Arctic has warmed four times faster than the global average^1–3^. This warming has led to diminishing snow and ice^4^, increased evaporation and precipitation^5^, thawing permafrost^6^ and other consequences for the natural ecosystems, living biotas and societies^7,8^. The warming has also driven increasing occurrences of extreme weather events such as persistent heatwaves^9,10^ or winter warming events^11^. Changes in temperature and precipitation have already caused major alterations in Arctic life and ecosystems^12,13^.

One of the most fundamental and profound changes has been the overall poleward increase in vegetation productivity, commonly referred to as “Arctic greening”^14–16^. However, the converse, i.e., “Arctic browning” has also occurred in some Arctic regions^14,17,18^. The climatic drivers of these biome-shift trends are multifarious and can be linked to baseline air temperature conditions as well as to both gradual climatic changes in bioclimate, e.g., growing season air temperature (greening or browning depending on the magnitude and direction of air temperature change), and sudden weather events, e.g., extreme winter warming (browning).

Critically, much current understanding of how Arctic terrestrial life and ecosystems will respond to climate change is based on data of long-term climate averages, such as the 30-year average climatologies, at coarse spatial resolutions of 10–100 km. However, short-term (intra- and interannual) bioclimatic variability linked with seasons and extreme weather events can exert a strong influence on the structuring and functioning of ecosystems^19^, and extreme events may have disproportionate impacts on ecosystems due to passing ecological or biological thresholds^20^. Thus, at the ecosystem level, climate change has manifested throughout trends in both annual and seasonal temperature and precipitation patterns, but also throughout extreme events, such as transient periods of extreme winter warmth, summer drought or high wind speeds. Such trends may only become apparent in daily or sub-daily climatic datasets extending over sufficiently long time periods.

Many scientific domains rely on high-quality climate information in an applicable format. For example, upscaling measurements of greenhouse gas fluxes^21^ or modeling of species distributions^22^ under past, current and future climates are typically carried-out on gridded climate datasets. Obviously, the realism of the outcome is partly dependent on the relevance of the used climate indices and the spatiotemporal resolution of the climate data.

Today, atmospheric reanalyses^23,24^ provide temporally and spatially consistent evolution on climate variables without being limited by the challenges arising from the uneven coverage of *in-situ* observations. Furthermore, by applying various downscaling methods, several spatially fine-grained bioclimatic datasets have been published in recent decades, e.g., WorldClim^25^, TerraClimate^26^, CHELSA^27^ or MERRAclim^28^. However, the climatologies traditionally presented – such as annual, seasonal, or monthly averages – focus mainly on seasonally-averaged temperature and precipitation, which may fail to capture many ecologically important aspects of the Arctic climate. For example, snow cover duration^29,30^, rain-on-snow events^31^, water vapor pressure deficit^32^ or extreme wind events^33^ are relevant for many biological, biogeochemical or geomorphological processes happening in the Arctic but may not be fully represented by the more commonly used climate statistics. Climate datasets important for the Arctic and its terrestrial ecosystems may therefore still lack the relevant temporal resolution or the relevant bioclimatic indices to better capture extreme events, and their recurrence and impacts.

**Fig. 1:**
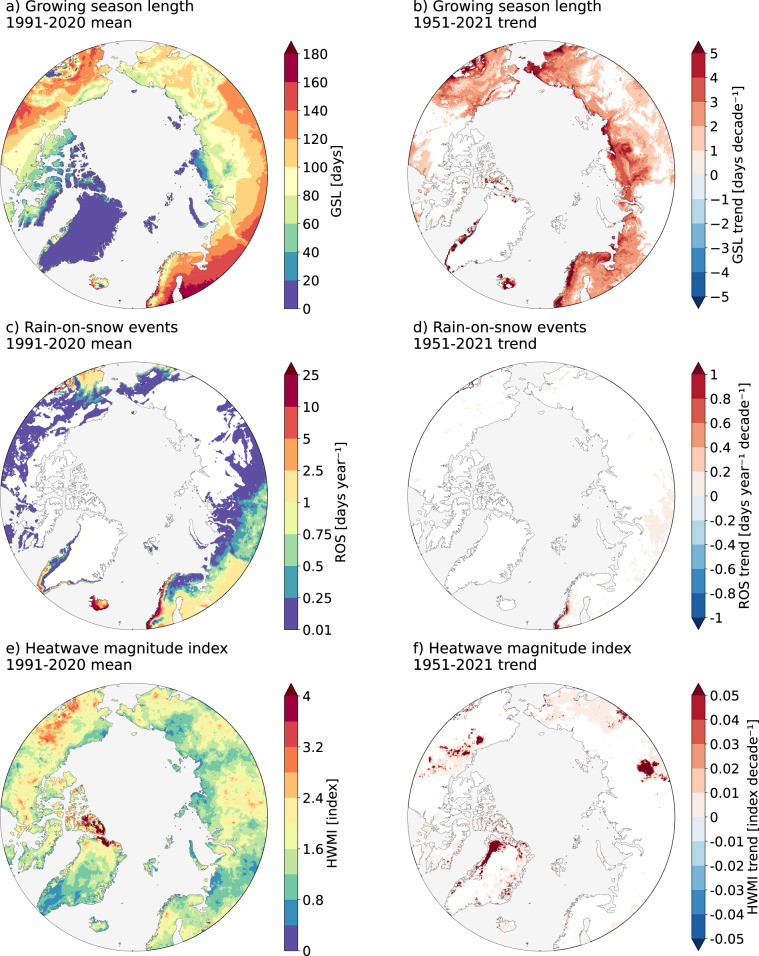
*Examples for 3 out of the 14 ARCLIM variables*. The left panels show the average conditions for the most recent climatological normal 1991–2020 and the right panels show the 1951–2021 linear trend in (**a,b**) the thermal growing season length (GSL), (**c,d**) the number of rain-on-snow events (considered from Nov-Apr period each year) and (**e,f**) the heatwave magnitude index. Note that the area shown in the maps extends north of 60°N, while the spatial domain of the dataset covers 45–90°N. The trends (**b,d,f**) are calculated using Theil-Sen slope estimator, and areas with statistically insignificant trends (p > 0.05, according to Mann-Kendall trend test) have been masked out (in white). The areas without rain-on-snow events (**c**) are shown with white color. See Methods section for the definitions of the variables.

Here, we present a new dataset of Arctic bioclimatic indices, called the Bioclimatic Atlas of the Arctic (ARCLIM). The ARCLIM dataset consists of 14 climate and event-type indicators that are particularly relevant for investigating the changes in the Arctic ecosystems driven by both trends (push) and stochastic events (pulse) accompanying climate change, such as growing season length, the number of rain-on-snow events or the heatwave magnitude index (Fig. 1). The ARCLIM variables are computed from the ERA5-Land dataset^34^, which includes global hourly data at 0.1 degree (i.e. 9 km) of spatial resolution for various surface parameters. The ARCLIM dataset covers the northern high-latitudes (45–90°N) from 1950 to 2021, hence providing a 72-year long consistent time series of seasonal climate and extreme event indicators in the Arctic. We provide ARCLIM variables representing: (1) the mean conditions for the climatology 1991–2020; (2) temporal trends over 1951–2021; and (3) annual values during 1950–2021.

## Methods

### The ERA5-Land dataset

The ARCLIM dataset is derived purely from the ERA5-Land dataset^34^. ERA5-Land, provided by European Centre for Medium-Range Weather Forecasts (ECMWF), is a downscaled re-simulation of the land component of global ERA5 reanalysis^23^. The horizontal resolution of ERA5-Land is 0.1°, downscaled from 0.25° used in ERA5. The ERA5-Land dataset is produced by the ECMWF land surface model Carbon Hydrology-Tiled ECMWF Scheme for Surface Exchanges over Land^35^, which is forced by the meteorological fields from ERA5. Observations are indirectly incorporated via comprehensive assimilation of instrumental and remote sensing observations into the ERA5 reanalysis dataset^23^. The ERA5-Land dataset is currently the most accurate modern reanalysis product in terms of horizontal and temporal resolution, making it a suitable dataset to use as a basis of the ARCLIM dataset. It is updated near real time, making it computationally feasible to update the ARCLIM dataset regularly.

### Downloading and pre-processing the input data

**Fig. 2:**
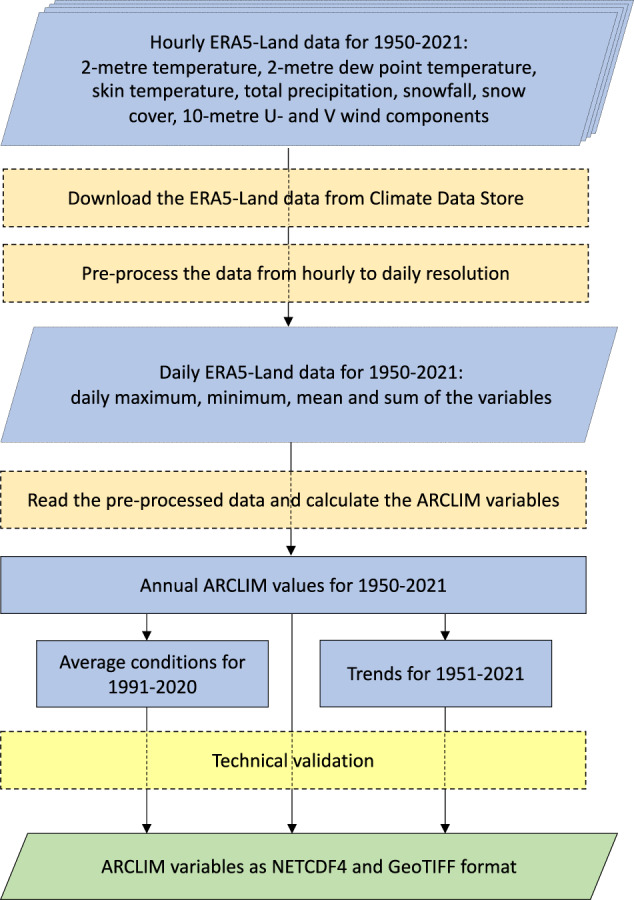
The production of the ARCLIM dataset step by step.

The ERA5-Land dataset was downloaded from Copernicus Climate Data Store (CDS, cds.climate.copernicus.eu) for the period 1950–2021^36,37^. The list of all downloaded input variables is shown in Table 1. The variables were downloaded for the domain north of 45°N, in the native 9-km spatial resolution and with a one-hour temporal interval (Fig. 2).

**Table 1:** List of input variables in the ERA5-Land dataset.

Full name	Short name	Unit	Pre-processing
2 metre temperature	2t	K	DMEA, DMAX, DMIN
2 metre dew point temperature	2d	K	DMEA, DMAX, DMIN
Skin temperature	skt	K	DMEA, DMAX, DMIN
Total precipitation	tp	m	DSUM
Snowfall	sf	m of water equivalent	DSUM
Snow cover	snowc	%	DMEA, DMAX, DMIN
10 metre U wind component	u10	m/s	DMEA, DMAX, DMIN
10 metre V wind component	v10	m/s	DMEA, DMAX, DMIN

DMEA = daily mean, DMAX = daily maximum, DMIN = daily minimum, and DSUM = daily sum.

Due to the large data volume of the hourly ERA5-Land dataset, for each day, we computed daily mean, maximum, minimum and sum values of the variables. These daily statistics were archived to CSC (Finnish IT center for science) Allas object storage system (Fig. 2). For the instantaneous variables (2t, 2d,skt, snowc, u10, v10), daily mean, maximum and minimum values were archived, and for the accumulated variables (tp, sf) only the daily sum values were archived. Furthermore, according to the ERA5-Land convention^34^, the accumulated variables are accumulated from 00 UTC to the next 24 hours (i.e., the accumulation at 00 UTC represents the accumulation during the previous day). Thus, before archiving tp and sf variables, we shifted the time axis backward by one day so that the accumulations at 00 UTC correspond to the actual day, and not the previous day.

### Calculation of ARCLIM variables from the pre-processed ERA5-Land dataset

For each year during 1950–2021, we read the archived daily ERA5-Land data from CSC Allas and calculated the set of ARCLIM variables (Table 2). The reading of archived ERA5-Land files and the calculation of ARCLIM variables were performed with Python programming language and standard packages, such as Xarray^38^. The calculation of ARCLIM utilised parallel computing with the Dask tool.

**Table 2:** The list of ARCLIM variables.

Full name	Abbreviation	Unit	Reference, if any	Input variable(s)
Thermal growing season length	GSL	days	Aalto *et al*.^42^	2t
Thermal growing degree day sum	GDD	°C days	Aalto *et al*.^42^	2t
Frost during the growing season	FGS	°C days		2t, skt
Freezing degree days	FDD	°C days		2t
Number of rain-on-snow events	ROS	year^−1^	Cohen *et al*.^51^	tp, sf, snowc
Number of winter warming events	WWE	year^−1^	Treharne *et al*.^55^	2t, snowc
Intensity of winter warming events	WWI	°C days	Treharne *et al*.^55^	2t, snowc
Heatwave magnitude index	HWMI		Dobricic *et al*.^10^	2t
Vapor pressure deficit magnitude index	VPDI			2t, 2d
Summer warmth index	SWI	°C	Berner *et al*.^62^	2t
Snow season length	SSL	days		snowc
Onset of snow season	SSO	Day of year		snowc
End of snow season	SSE	Day of year		snowc
Number of high wind speed events	HWE	year^−1^		u10, v10
Annual mean temperature	TAVG	K		2t
Annual precipitation	PRA	m		tp
Annual snowfall	SFA	m		sf
Annual 10-m wind speed	WSA	ms^−1^		u10, v10

Further details and definitions are provided in the main text.

The calculations of the ARCLIM variables were done in the native 0.1° by 0.1° regular latitude-longitude ERA5-Land grid. Thus, no spatial interpolation was performed in any stage of the production of the ARCLIM dataset. As ERA5-Land is a land-only dataset, the ARCLIM variables were calculated only for terrestrial areas. Therefore, the grid cells representing the ocean are marked as missing values and are displayed as NaN in the Xarray datasets.

After computing the annual values for each year during 1950–2021, we calculated the average conditions for the period 1991–2020 and the trends for 1951–2021 for each variable (Fig. 2). The magnitude and statistical significance of the trends were calculated using the Theil-Sen slope estimator^39,40^ and Mann-Kendall trend test, respectively, as implemented in the *pyMannKendall* Python package^41^. The p-values representing the statistical significance of the trends are provided in the dataset. The trends are calculated starting from 1951, because 1951 is the first complete year representing the winter conditions in the ERA5-Land dataset.

Below, we provide short descriptions of the ARCLIM variables. The maps depicting both the average conditions of 1991–2020 and the trends over 1951–2021 for each variable can be found from the supplementary material (Figs. S1–S18). In addition to the 14 listed bioclimatic variables, ARCLIM includes annual mean 2-m temperature, annual precipitation, annual snowfall and annual mean 10-m wind speed (Table 2).

#### Thermal growing season length (GSL)

The period of the year when daily mean temperature remains permanently at or over a predefined threshold. In agreement with several previous studies^42–45^, 5 °C is used as a threshold. The beginning and the end of the growing season are determined using the integral method^46^. The integral method identifies the local minimum of the summation curve of T - 5 °C, where T is the daily mean temperature in degrees Celsius. The day subsequent to this minimum is the first day of the growing season. Likewise, the absolute maximum of the sum T - 5 °C is used to determine the end date of the growing season. Moreover, the onset date must be earlier than the 1st of July, otherwise the growing season is not defined. Productivity and life cycles of Arctic organisms are limited not only by the overall temperatures but also by the length of the short growing season^47^. Thus, even a minor absolute lengthening of the thermal growing season can induce profound consequences in relative terms in the Arctic.

#### Thermal growing degree day sum (GDD)

The sum of daily mean temperatures which exceed the 5 °C threshold during the growing season. The growing season is defined as above by the GSL variable. Temperatures below the threshold within the growing season do not decrease the sum. Thermal growing degree day sum integrates the length and overall warmth of the warm season and is a commonly used variable to characterize the overall summer thermal conditions in ecological modeling of cold ecosystems^46,48^.

#### Frost during the growing season (FGS)

The sum of daily minimum skin temperatures which are below freezing during the growing season, as defined by GSL. Skin temperature is extracted from ERA5-Land dataset and represents the temperature of the uppermost surface layer of the Earth. The frost sum is positive and sums negative temperature values, e.g., a skin temperature of −4 °C increases the sum by 4 °C. Arctic plants and other organisms can generally tolerate cold temperatures, but they are typically most sensitive to frost damage during the early flowering season when reproductive organs are exposed^49^.

#### Freezing degree days (FDD)

The sum of daily mean temperatures which are below freezing during the winter season. The onset of the freezing season is determined by the integral method (i.e., the local maximum in the summation curve of daily mean temperatures). The end of the freezing season is determined using the local minimum of cumulative daily mean temperatures. The year denotes the year of the late winter season (e.g., freezing season 2020 refers to the period of July 2019–June 2020). The freezing degree day sum is positive and sums negative temperature values, e.g., a daily mean temperature of −4 °C increases the sum by 4 °C. Freezing degree day summarizes the ‘harshness’ of winter conditions relevant for organisms exposed to free atmosphere conditions without the shelter of snow cover^50^.

#### Number of rain-on-snow events (ROS)

Rain-on-snow (ROS) events are defined as days with total liquid precipitation greater than 5 mm on a snow-covered grid cell. A snow-covered grid cell is defined as a grid cell whose daily averaged snow cover fraction is 0.5 or greater. Daily liquid precipitation is obtained by subtracting snowfall from the total precipitation. The definition is based on a combination of definitions by Cohen *et al*.^51^ and Mooney and Li^52^. All ROS events are considered from the whole winter (Nov-Apr) season. The year denotes the year of the late winter season (e.g., ROS events in 2020 refers to the period from November 2019 to April 2020). ROS events can drastically affect the properties of snow pack, soil temperatures, cause surface icing and thus damage plants and affect feeding of Arctic mammals^31,53,54^.

#### Number of winter warming events (WWE)

Adapted from Treharne *et al*.^55^, winter warming events (WWEs) are defined as days when daily mean temperature of 2 °C or higher occurs in a grid cell which is snow-covered. A snow-covered grid cell is defined as a grid cell whose daily averaged snow cover fraction is 0.5 or greater. In line with Treharne *et al*.^55^, WWEs are calculated only from the November-April period to avoid these extreme events being confounded with warm periods towards the end of autumn or start of spring. The year denotes the year of the late winter season (e.g., WWEs in 2020 refers to the period of November 2019 until April 2020). The so-called WWEs can melt the insulating snowpack, lead to premature loss of winter freeze tolerance in plants and thus, cause frost damage in vegetation if followed by cold temperatures^55,56^.

#### Intensity of winter warming events (WWI)

To better capture the biological importance of WWEs, a further intensity metric was used. The intensity of WWEs is defined according to Treharne *et al*.^55^. The cumulative daily mean air temperature (°C) is linearly weighted by the duration throughout the WWE: for example, for a 3-day event with daily mean air temperatures of 4 °C, 6 °C and 3 °C, the intensity is defined as (4 * 1) + (6 * 2) + (3 * 3) = 25 °C days. This is used to capture the increasing importance of duration in initiating biological responses (such as loss of cold tolerance in plants). The annual intensity is the total accumulated intensity of the events within a given winter. The events are calculated only from the November-April period. The year denotes the year of the late winter season (e.g., WWEs in 2020 refer to the period of November 2019 until April 2020).

#### Heatwave magnitude index (HWMI)

This indicator is calculated according to the definition presented in earlier studies^10,57^. The index is based on daily maximum 2-m temperature, and its calculation consists of the following stages:

1. For each day of the June-August period, we calculate the 90th percentile of daily maximum temperatures using a 31-day moving window and the 1981–2010 climatology. This means a distribution of 930 values (31 days × 30 years) for each summer day from which the 90th percentile is calculated.
2. Heatwave days are then defined as days during the June-August period when the daily maximum temperature exceeds the predefined 90th percentile for that calendar day. The heatwave needs to last at least three consecutive days. Hence, events which last less than three days are excluded from the computation.
3. Based on Dobricic *et al*.^10^, the daily magnitude of the heatwave is defined as *MD* = (*T*_*d*_-*T*_25_)/(*T*_75_-*T*_25_), where *T*_*d*_ is the daily maximum temperature, and *T*_75_ and *T*_25_ are the 75th and 25th percentiles, respectively, of annual maximum temperature during the 1981–2010 period (30 values). Thus, if the daily maximum temperature equals the 75th percentile, *Md* gets a value of 1.
4. The magnitude of each separate heatwave in a given summer is defined as the cumulative sum of daily heatwave magnitudes (step 3 above) during the heatwave. There may be several heatwaves in the grid cell within a given summer, so the cumulative values are calculated separately for each heatwave.
5. Finally, the heatwave magnitude index for a given grid cell is defined as the maximum cumulative magnitude of the strongest single heatwave occurring within a given summer.

Intensive heatwaves can cause unprecedented melting rates in the Arctic ice sheets and rapid permafrost thawing, intensified wildfires and drought that can damage the Arctic plants not adapted to high temperatures^10^.

#### Vapor pressure deficit magnitude index (VPDI)

This indicator represents high vapor pressure deficit (VPD) events during the summer period (June-August). The VPD variable is calculated from the difference between the saturated vapor pressure and the actual vapor pressure, as follows:

$$VPD=VPsat-VPair,$$

where *VPsat* is the saturated vapor pressure (in Pa), calculated with improved Magnus formula^58^.

$$VPsat=610.94\ast {e}^{\left(\left(17.625\ast T2m243.04+T2m\right)\right)},$$

and *VPair* is the actual vapor pressure (or the vapor pressure at dew point temperature):

$$VPair=610.94\,\ast \,{e}^{\left(\left(17.625\ast D2m\right)/\left(243.04+D2m\right)\right)}.$$

In these equations, *T*2*m* and *D*2*m* are the daily-averaged 2-m temperature and 2-m dew point temperature in Celcius, respectively. The vapor pressure magnitude index (VPDI) is calculated following the methodology of heatwave magnitude index (see above), but using VPD instead of the daily maximum temperature. Thus, the VPDI represents the magnitude of the strongest single VPD event within a given summer. Increasing VPD influences plants’ stomatal conductance and transpiration and thus has subsequent impacts on photosynthesis and growth with higher risks of carbon starvation and desiccation^59,60^. In addition, increasing VPD, through reduced transpiration, reduces the buffering effect of vegetation on local microclimatic conditions^61^.

#### Summer warmth index (SWI)

Summer warmth index (SWI) is calculated as the annual sum of monthly mean 2-m temperatures above 0 °C. The monthly mean 2-m temperatures are derived from daily mean 2-m temperatures. The SWI is commonly used as an indicator of cumulative summer heat load in the Arctic^62–64^, and the recently increased summer air temperatures have been shown to explain large parts of the observed greening across the Arctic tundra^62^.

#### Snow season length (SSL)

The length of the snow season (SSL) is defined as the longest continuous period of the year when the grid cell is snow-covered (i.e., the daily averaged snow cover fraction is 0.5 or greater). The year denotes the year of the late winter season (e.g., snow season 2020 refers to the winter 2019/2020). Besides temperature, snow cover duration limits the time of activity and availability of food resources for most of the Arctic terrestrial organisms^65^. Changes in snow cover duration may alter the energy balance of the whole Arctic^66^ but also cause major shifts in surface-level microclimates^67^.

#### Onset of snow season (SSO)

The onset of the snow season (SSO) indicates the first day of the snow season, as defined by the variable SSL. The day is given as the ordinal day of the year (1–366). In those cases when the onset of the snow season occurs after the New Year, the day numbering continues after 365/366 (e.g. January 1 = 366, January 2 = 367,…). This procedure allows that the variable becomes continuous and there are no step changes in the time series after day 365. At latest the SSO determines the start of dormancy for low-growing vegetation^65^. Because snow effectively insulates the ground, timing of the SSO in relation to temperature fluctuations is important in determining the soil temperatures of early winter and is thus related, e.g., to soil microbial activity and permafrost^6,68^.

#### End of snow season (SSE)

Similarly as SSO, the end of the snow season (SSE) indicates the day of the year when snow season ends, as defined by the variable SSL. The day is given as the ordinal day of the year (1–366). In those cases when the end of the snow season occurs before the New Year, the day numbering goes negative (e.g., December 31 = 0, December 30 = −1, etc.). The SSE variable is strongly related to phenology (i.e., the timing of life events) of Arctic organisms and can be one of the most important factors limiting the length of the growing season as snow can still be present long after the above air has already warmed up^66,69^.

#### Number of high wind speed events (HWE)

This indicator represents the annual number of days when the 10-m height daily maximum wind speed in the grid cell exceeds the predefined threshold. The threshold is calculated using a 31-day moving window and the 1981–2010 climatology of daily maximum wind speed. This means a distribution of 930 values (31 days × 30 years) for each calendar day from which the 90th percentile is calculated. The wind speed in the grid cell is calculated from the daily maximum 10-m u- and v-wind components as:

$$ws=\sqrt{u1{0}^{2}+v1{0}^{2}}$$

High wind speed relocates snow, organic matter and fine sediments and distributes seeds and spores of plants^70,71^ but may also cause desiccation and wind-chill to Arctic organisms^72–74^.

## Data Records

The ARCLIM dataset is available through Figshare repository^75^. The dataset consists of three subsets: (1) the annual values for 1950–2021; (2) the average conditions for the 1991–2020 climatology; and (3) trends and their associated p-values over 1951–2021. Note that the annual values (1) are aggregated over the whole year or over a specific time period of the year (e.g., growing season, winter season, etc.). Thus, for event-type variables, ARCLIM does not provide information on when a single event during the year has occurred. This information would require a daily temporal resolution that would multiply the volume of the data and was therefore not possible to provide in the public repository.

The datafiles are provided both in a NETCDF4 and GeoTIFF format. The files for annual values follow the naming format arclim_variable, where “variable” refers to the abbreviation shown in Table 2 (e.g., arclim_GSL.nc or arclim_GSL.tif). The averages for the 1991–2020 climatology for all variables are in the file named arclim_means.nc, and analogously trends and p-values in the files named arclim_trends.nc and arclim_pvalues.nc, respectively.

## Technical Validation

The ARCLIM dataset is derived from ERA5-Land, which is a state-of-the-art global dataset for various land applications. As ERA5-Land is still a relatively new dataset, only a few studies so far have validated its performance against *in-situ* observations.

Regarding snow depth, Muñoz-Sabater *et al*.^34^ documented that ERA5-Land performs varyingly compared to ERA5. The ERA5-Land dataset outperforms ERA5 in the US and in complex terrain where ERA5-Land benefits from its higher spatial resolution. Instead, in Scandinavia, ERA5 was found to perform better than ERA5-Land. Räisänen^76^ compared mean snow depth in March for 39 winter seasons (1982–2020) from ERA5-Land predictions with two Finnish station observations: Sodankylä and Helsinki. He found high interannual correlation (0.92–0.97), but a slight negative bias for Sodankylä (i.e., observations had less snow than predicted by ERA5-Land).

For precipitation, Xu *et al*.^77^ showed that ERA5-Land and ERA5 exhibit similar performance in precipitation metrics on an annual basis, but both datasets were found to underestimate the mean rate of precipitation events over China. Given that total precipitation from ERA5 is used as an atmospheric forcing field in the production of ERA5-Land, their similar characteristics in precipitation metrics is perhaps not surprising.

In Finland, winter (November-March) precipitation from ERA5-Land were found to be overestimated by 12% in Helsinki and by 18% in Sodankylä^76^ compared to observations, but these discrepancies may be explained by the rain gauge undercatch which is prominent, especially in winter, when precipitation is mostly solid^78^. Despite these slight biases in precipitation, Räisänen^76^ reported high interannual correlation (0.91–0.96) between ERA5-Land predictions and precipitation observed at meteorological stations in winter, even though ERA5 (and thus ERA5-Land) does not assimilate precipitation observations from Europe^23^.

To further assess the performance of the ERA5-Land dataset, we conducted a validation for summer (June-August) and winter (December-February) mean temperatures at 2-m height (T2m) using station-based data from the Global Historical Climatology Network Monthly (GHCN-M) database version 4^79^. We used the station data which was homogenized (indicated by the suffix “.qcf” in the GHCN-M database) for non-climatic effects such as station relocations or time-of-observation biases. The homogenization was also the reason why we did not use daily station data for validation because the daily observations in GHCN-Daily are not homogenized^80^.

**Fig. 3:**
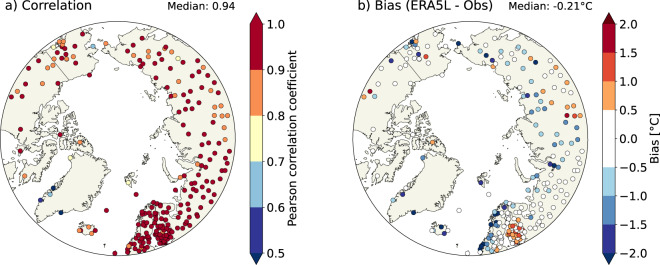
Validation of summer temperatures. Pearson correlation coefficient (**a**) and bias (**b**) in summer (June-August) mean temperatures between ERA5-Land predictions and GHCN-M observations. The bias is defined as ERA5-Land predictions minus GHCN-M observations. The statistics are calculated only for stations located north from 60°N and with at least 60 years of data during the 1950–2021 period.

**Fig. 4:**
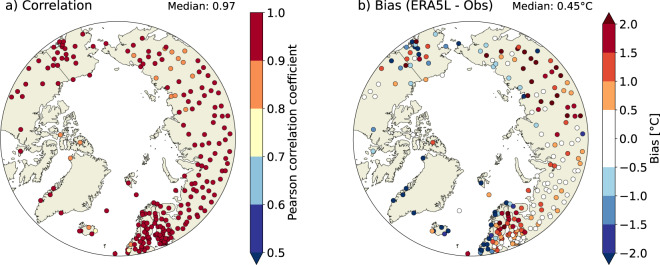
Validation of winter temperatures. Same as Fig. 3, but for winter (December-February) mean temperatures.

As ARCLIM is primarily intended for monitoring the Arctic bioclimate and studying responses of northern land ecosystems to climate change, the validation was restricted for the areas north of 60°N. We calculated the average temperatures for summer (June-August) and winter (December-February) from both weather station observations and ERA5-Land predictions. Only those weather stations that had complete data for at least 60 years during the 1950–2021 period were included in the validation. The ERA5-Land data corresponding to the weather station locations were extracted using the grid cells that contain the location of the weather stations. We noted that part of the stations located at the coastline could not be used, as the nearest ERA5-Land grid point was located in the sea and therefore no data was available for those locations. In total, our criteria resulted in 231 weather stations for the summer period (Fig. 3) and 237 weather stations for the winter period (Fig. 4). We calculated Pearson correlation coefficient (R), the bias (defined as the average difference: ERA5-Land predictions minus weather stations’ observations), root mean square error (RMSE) and the slope of the regression for each weather station. These results are discussed in the subsequent paragraphs.

The observed interannual variability in summer temperatures are reproduced relatively well in ERA5-Land (Fig. 3a). The median correlation (R) across all 237 weather stations is 0.94 (Fig. 3a; Table 3), with 80% of the weather stations reporting R > 0.90. The correlation appears to be the highest over Fennoscandia and western Russia. These regions also have the densest observation network, which may partly explain the higher correlation, due to the larger number of assimilated observations to ERA5. In line with the highest correlation, RMSE is the lowest over Finland and western Russia (Fig. S19). In contrast, ERA5-Land performs the least well for the few stations located on the coast of Greenland, which all have R < 0.8. The median bias (−0.21 °C, Fig. 3b) is negative, implying that summer temperatures in ERA5-Land tend to be slightly lower than the values measured at the weather stations. The most negative biases are associated with the weather stations located near the coasts, in particular along the Norwegian coastline.

**Table 3:** Validation statistics between weather station observations and predicted ERA5-Land values.

	Median correlation	Median bias	Median RMSE	Median slope
Summer (Jun-Aug)	0.94 (0.83, 0.98)	−0.21 (−2.07, 0.93)	0.67 (0.32, 2.22)	1.03 (0.83, 1.21)
Winter (Dec-Feb)	0.97 (0.88, 0.99)	0.45 (−3.89, 2.63)	1.17 (0.56, 4.41)	0.93 (0.77, 1.10)

The values are medians across all 231 (summer) and 237 (winter) weather stations. The bias is defined as the average difference: ERA5-Land predictions minus weather stations’ observations. The values in the parentheses represent the 5th and 95 h percentiles of the distributions.

In winter, ERA5-Land performs better than in summer (Fig. 4). The correlation is R > 0.90 for 91% of the stations, with a median correlation across all stations of R = 0.97 (Fig. 4a, Table 3). The higher correlation in winter than in summer can potentially be explained by the higher interannual variability of winter temperatures than summer temperatures. In contrast to the negative bias in summer, the bias is mostly positive in winter (Fig. 4b). The warm bias is prominent especially in the interior of Siberia, which is a region known for its cold winters. These regions also have higher RMSE (Fig. S20). Earlier studies have demonstrated that numerical weather prediction models^81^ and reanalyses^82–84^ struggle to accurately simulate strong stable boundary layers (SBL), and therefore tend to have warm bias especially in the cold-season Arctic^83^. Thus, our findings of positive winter T2m-bias in ERA5-Land (Fig. 4b) potentially stems from SBL-issues in ERA5. Nevertheless, similar to the negative summer bias, the overall positive bias in winter appears to be mostly negative in the coastal stations of the European sector of the Arctic (Norway, Svalbard, Iceland and Greenland).

Comparing gridded predictions with weather station observations is not always meaningful due to inherent mismatches in the scale of the data being compared. The temperature in the ERA5-Land represents the average conditions of each 0.1° grid cell (about 52 km² at 65°N), and is affected by the average elevation of the grid cell. Instead, the station observations are point-based. Therefore, in complex terrain, such as in Norway or Greenland, the average elevation of the grid cell may considerably differ from the station elevation. We found that especially in summer, the large negative biases in temperature are largely explained by the differences in the elevation (Fig. S21a). In winter, the elevation biases were found to be a less explanatory factor (Fig. S21b), which is in line with our assumption that issues in simulating winter SBL may play a larger role in the winter temperature biases.

The biases in ERA5-Land affect those ARCLIM variables which are calculated using absolute temperature thresholds. For example, the negative summer T2m-bias in some coastal Arctic stations (Fig. 3b), where the daily mean temperature barely rises above 5 °C threshold during summer, means that GDD and GSL may not be possible to compute in ARCLIM. However, as noted earlier, the grid cell in ARCLIM represents a wider area and thus the direct comparison to the *in-situ* observations is problematic. Nevertheless, given the high interannual correlation in both summer (R = 0.94) and winter (R = 0.97) temperatures, we consider that the ARCLIM dataset largely captures the interannual variability of the bioclimatic variables, especially those which are temperature-related.

## Supplementary information

Supplementary information for “Bioclimatic atlas of the terrestrial Arctic”

## Data Availability

The Python codes needed to reproduce the dataset are available from Github: https://github.com/fmidev/resiclim-climateatlas.
