# Supplementary material for: Bioclimatic atlas of the terrestrial Arctic
Source: Sci Data. 2023 Jan 19;10:40. doi: 10.1038/s41597-023-01959-w (PMC9852483; doi:10.1038/s41597-023-01959-w)
Supplement: Supplementary file 1 — Supplementary information for “Bioclimatic atlas of the terrestrial Arctic” [file 41597_2023_1959_MOESM1_ESM.pdf]

Supplementary information for “Bioclimatic atlas of the terrestrial Arctic”

Mika Rantanen[1]*, Matti Kämäräinen[1], Pekka Niittynen[2,6], Gareth K Phoenix[3], Jonathan Lenoir[4], Ilya Maclean[5], Miska Luoto[6] and Juha Aalto[1, 6]

[1]Finnish Meteorological Institute, Helsinki, Finland
[2]Department of Biological and Environmental Science, University of Jyväskylä
[3]Plants Photosynthesis and Soil, School of Biosciences, University of Sheffield, Sheffield, S10 2TN, United Kingdom
[4]UMR CNRS 7058, Ecologie et Dynamique des Systèmes Anthropisés (EDYSAN), Université de Picardie Jules Verne, Amiens, France
[5]Environment & Sustainability Institute, University of Exeter Penryn Campus, Penryn, TR10 9FE, United Kingdom
[6]Department of Geosciences and Geography, University of Helsinki, Helsinki, Finland

Supplementary figures

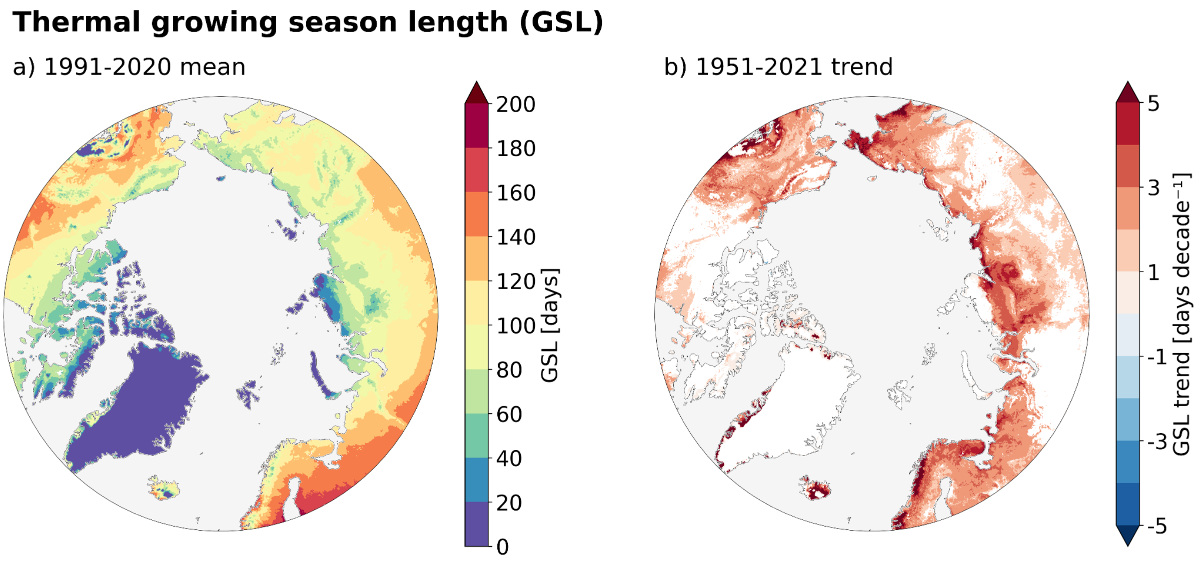

Figure S1. Growing season length a) average for 1991-2020 and b) trend for 1951-2021. The trends in b) have been calculated using Theil-Sen slope estimator, and areas with statistically insignificant trends (p>0.05) have been masked out.

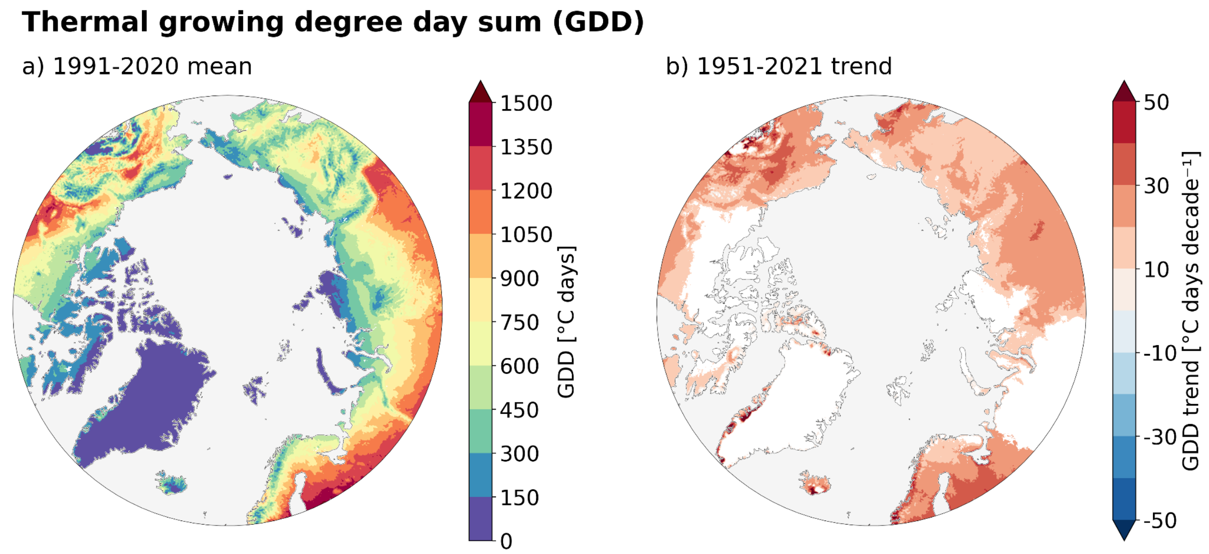

Figure S2. Same as Fig. S1, but for the growing degree day sum.

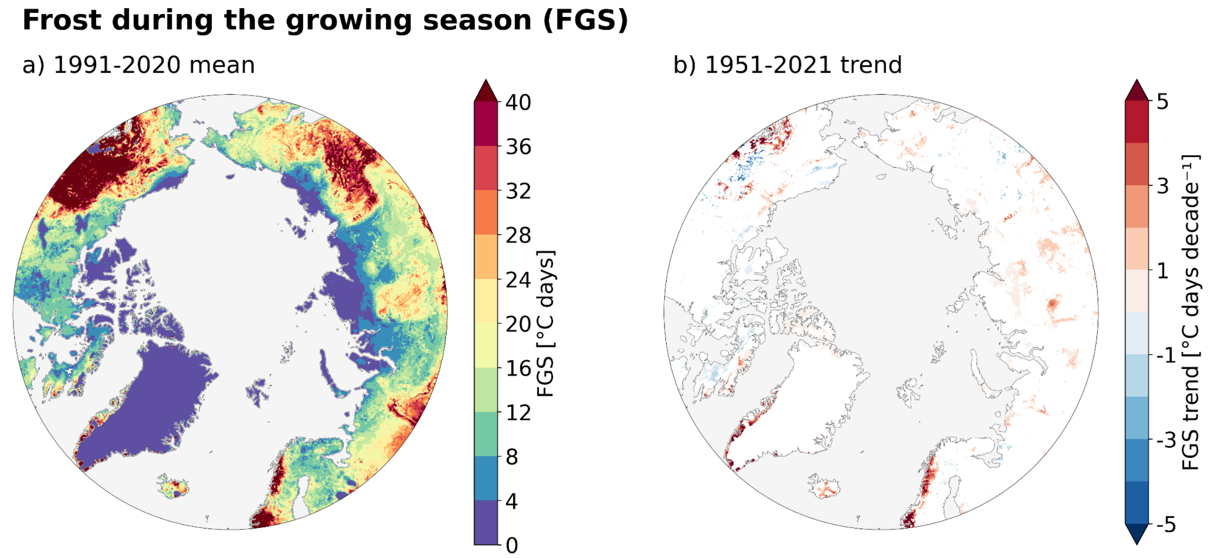

Figure S3. Same as Fig. S1, but for frost during the growing season.

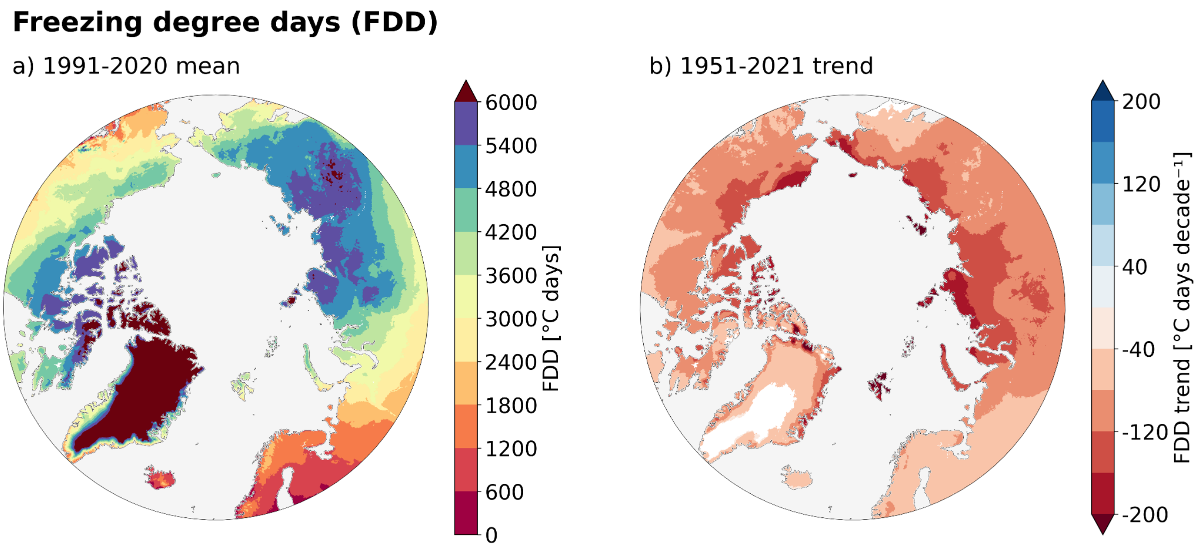

Figure S4. Same as Fig. S1, but for freezing degree days.

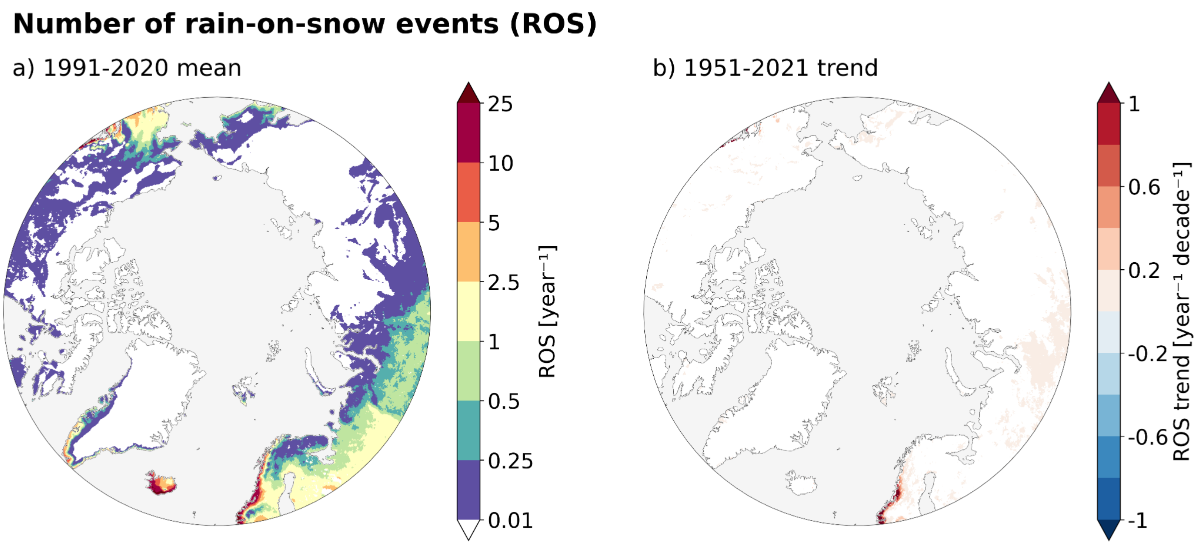

Figure S5. Same as Fig. S1, but for the number of rain-on-snow events.

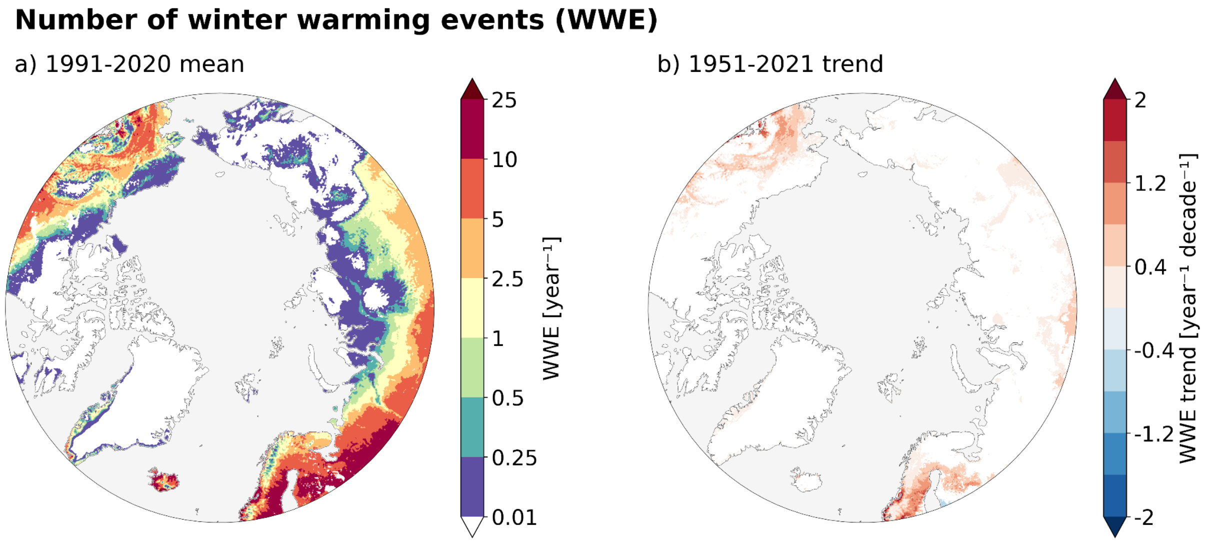

Figure S6. Same as Fig. S1, but for the number of winter warming events.

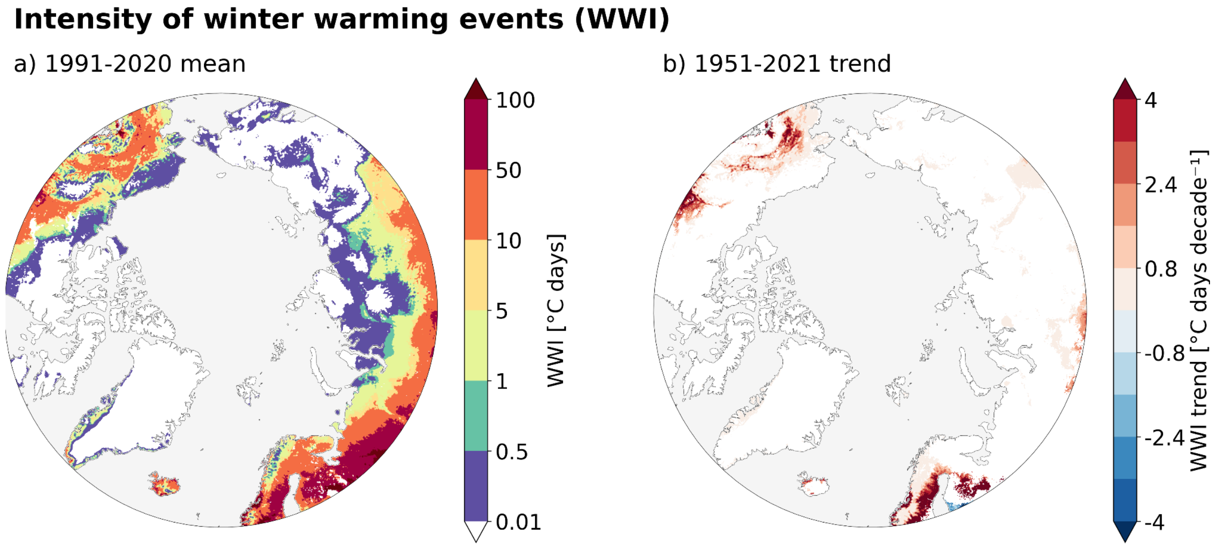

Figure S7. Same as Fig. S1, but for the intensity of winter warming events.

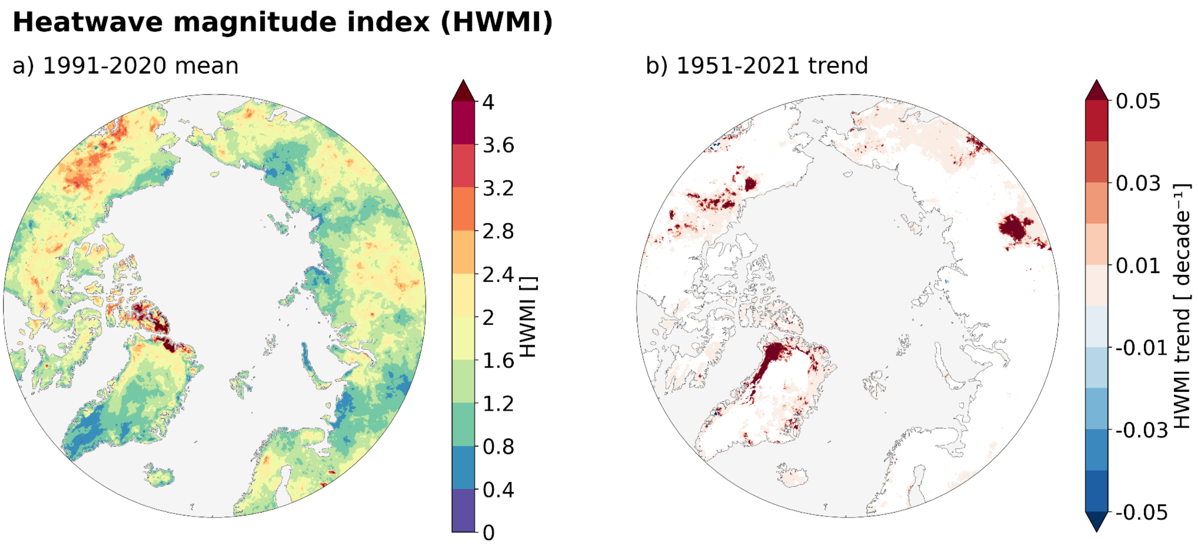

Figure S8. Same as Fig. S1, but for the heatwave magnitude index.

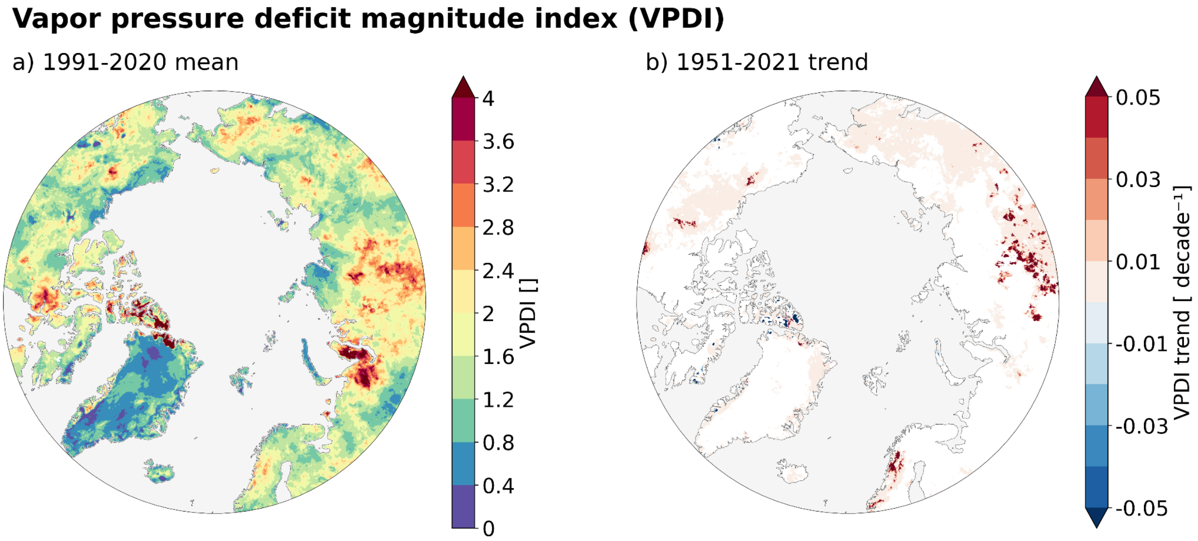

Figure S9. Same as Fig. S1, but for the vapor pressure deficit magnitude index.

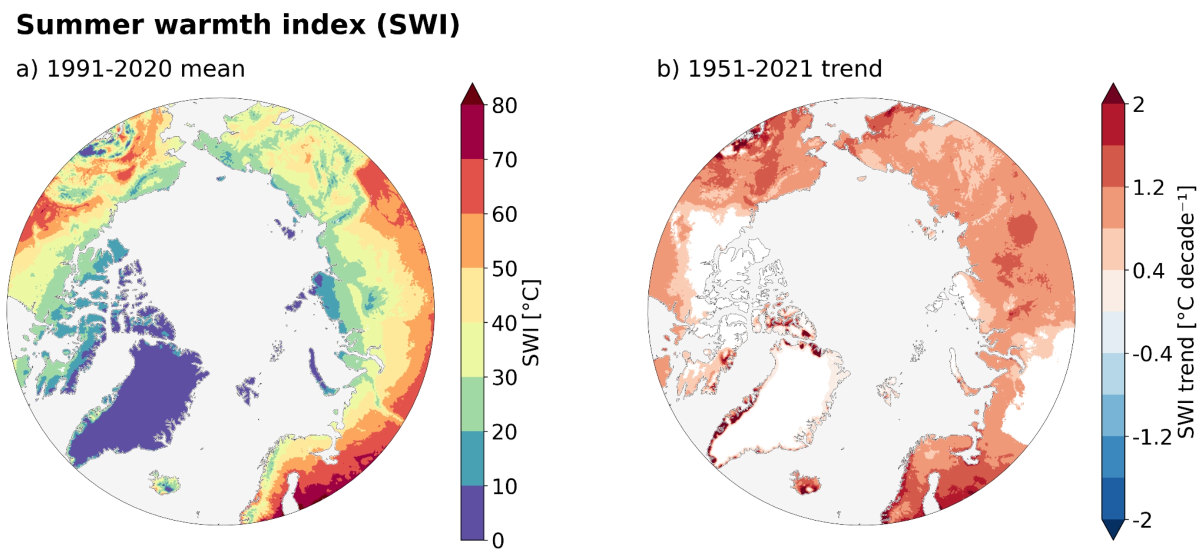

Figure S10. Same as Fig. S1, but for the summer warmth index.

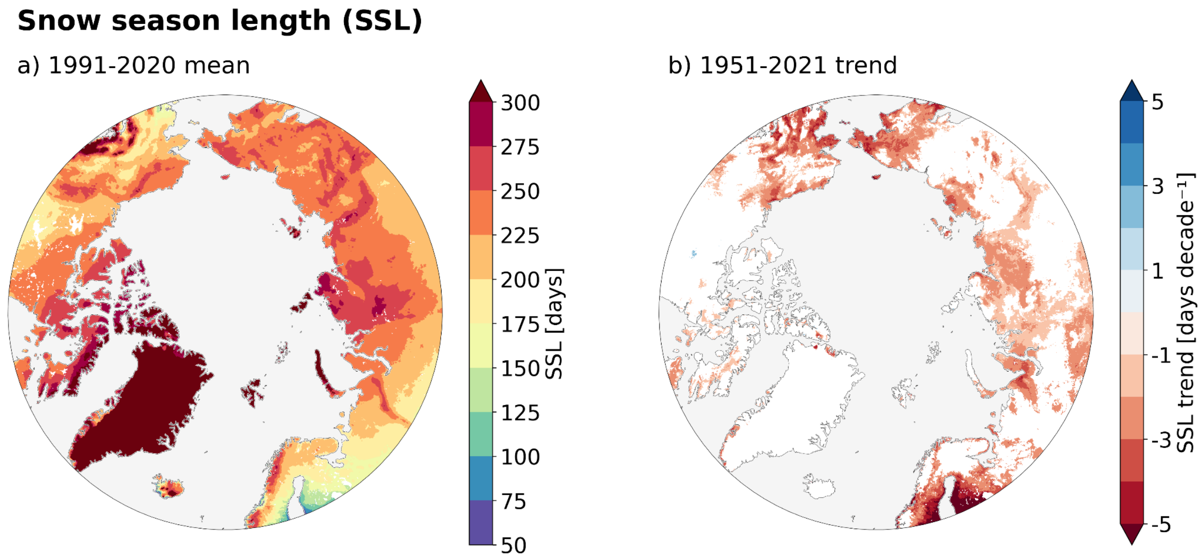

Figure S11. Same as Fig. S1, but for the snow season length.

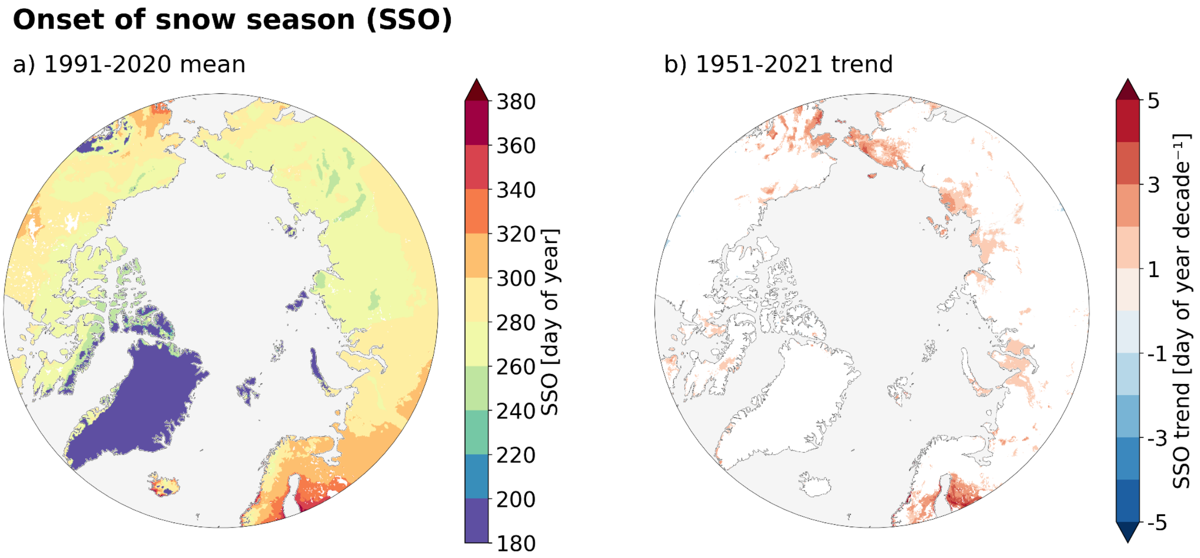

Figure S12. Same as Fig. S1, but for the onset of snow season.

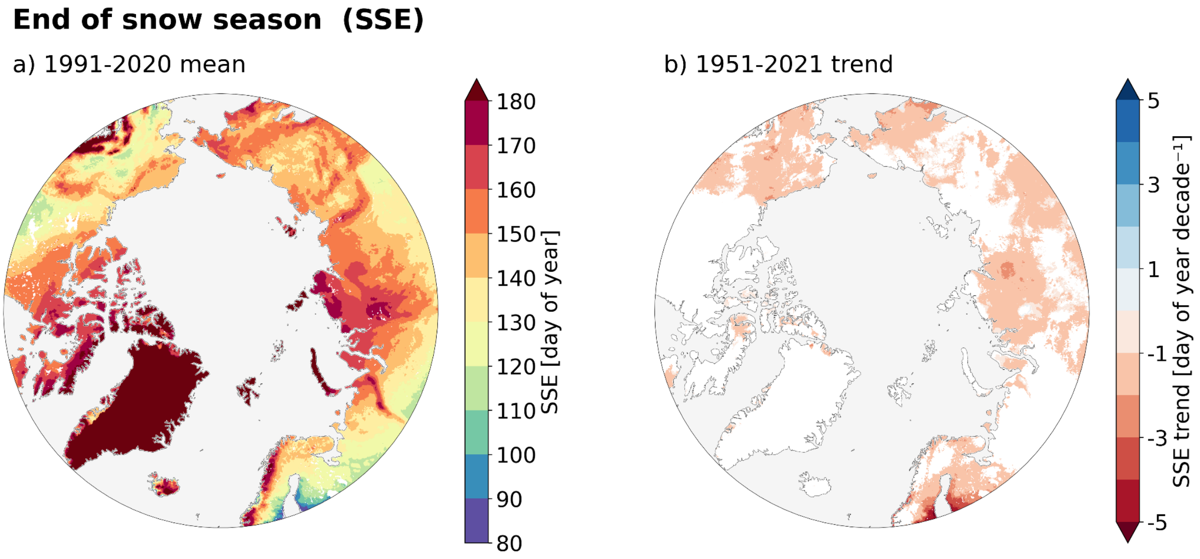

Figure S13. Same as Fig. S1, but for the end of snow season.

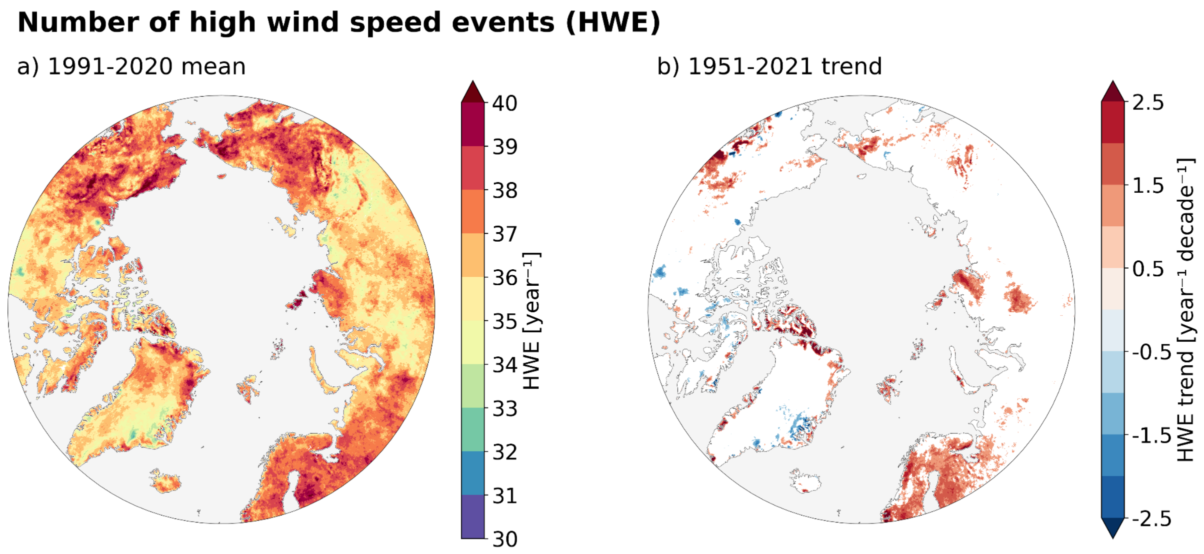

Figure S14. Same as Fig. S1, but for the number of high wind speed events.

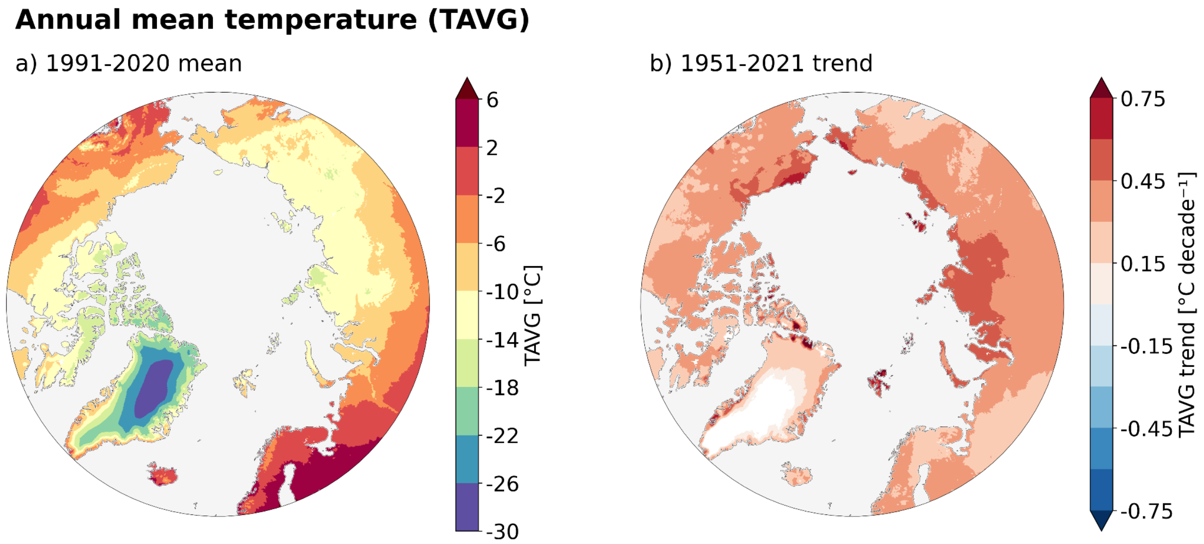

Figure S15. Same as Fig. S1, but for the annual mean temperature. Note that the temperature is visualized with Celcius but is given as Kelvin in the datafiles.

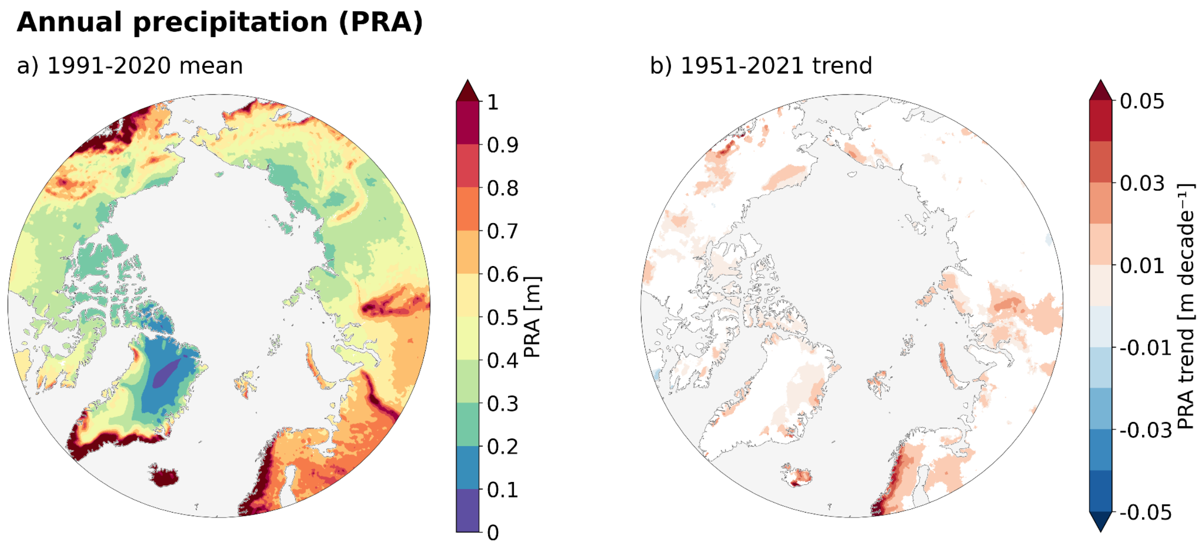

Figure S16. Same as Fig. S1, but for the annual precipitation.

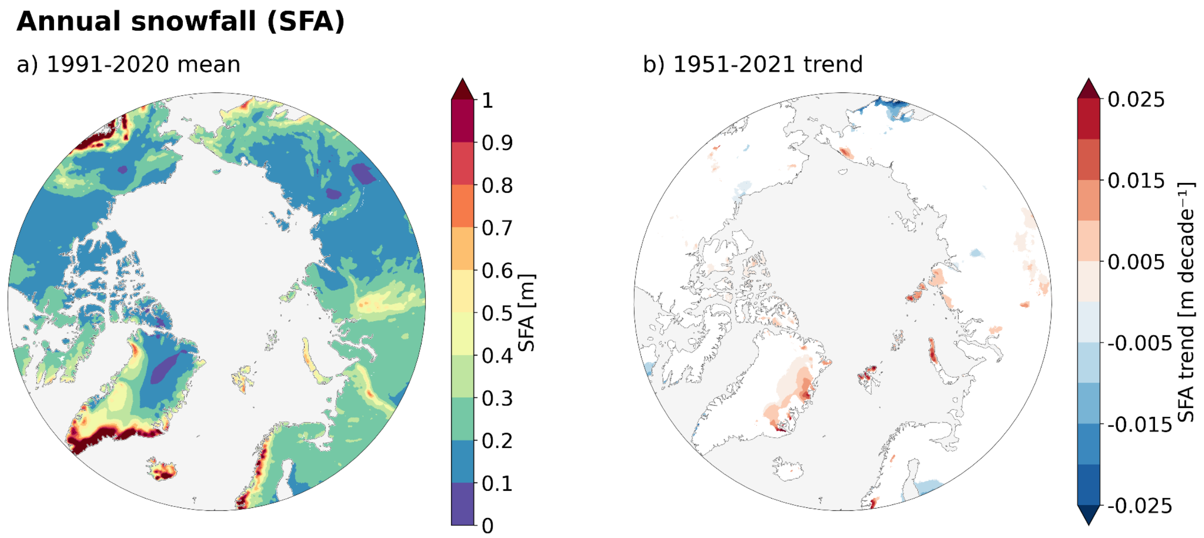

Figure S17. Same as Fig. S1, but for the annual snowfall.

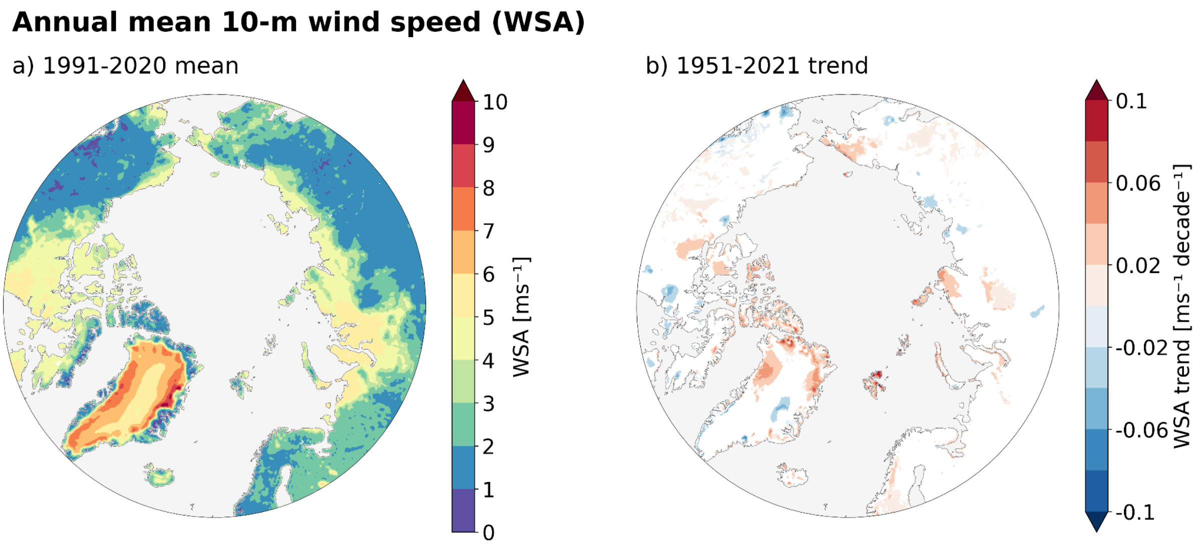

Figure S18. Same as Fig. S1, but for the annual 10-metre wind speed.

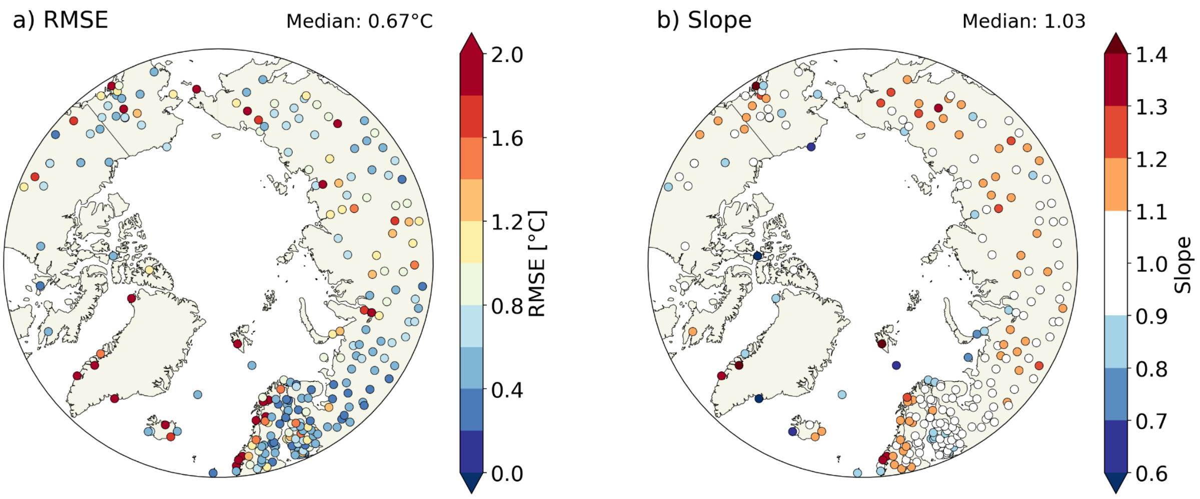

Figure S19. Root-mean-square error (a) and the slope of the regression (b) in summer (Jun-Aug) mean temperatures between ERA5-Land predictions and GHCN-M observations. The statistics are calculated only for stations located north from 60°N and with at least 60 years of data during the 1950-2021 period.

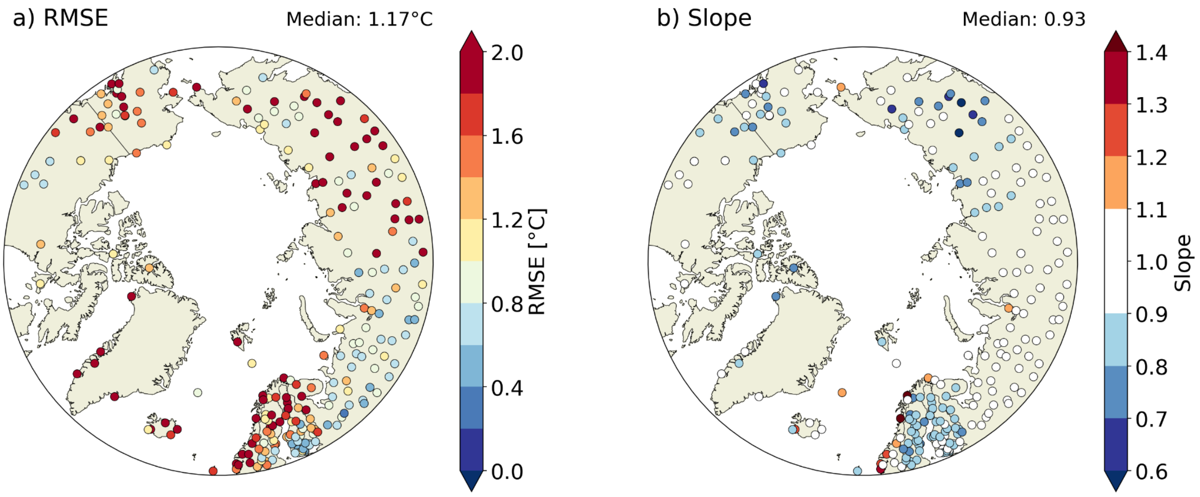

Figure S20. Same as Fig. S18, but for winter (Dec-Feb) mean temperatures.

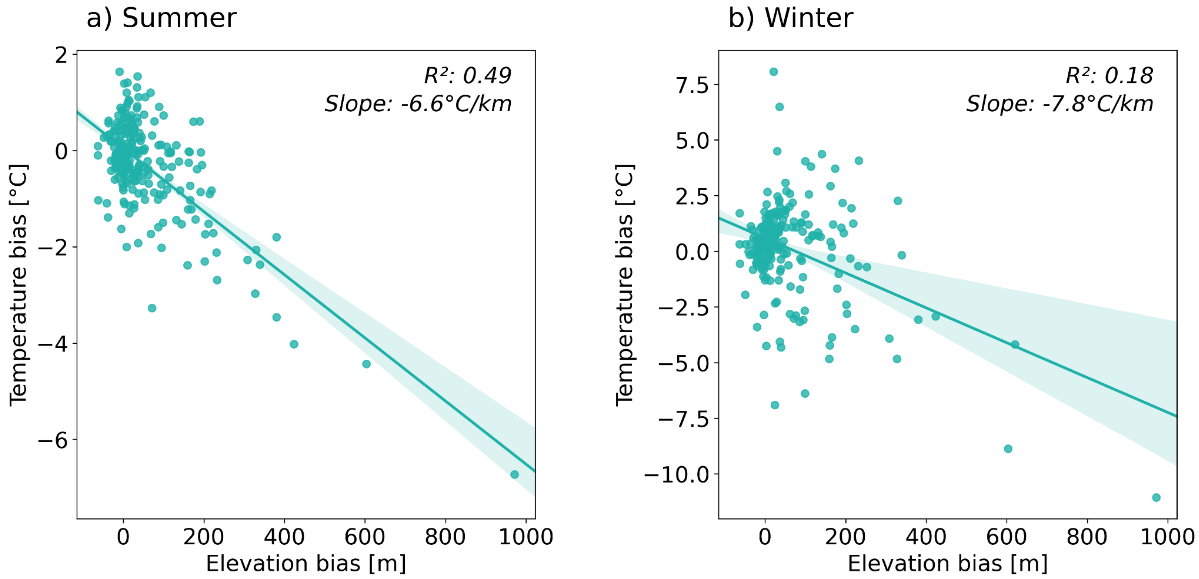

Figure S21. The effect of elevation bias (x-axis) on the temperature bias (y-axis) in summer (a) and winter (b). The elevation bias is defined as ERA5-Land grid cell elevation minus station elevation, and the temperature bias is defined as ERA5-Land mean temperature minus station mean temperature. Note the different y-axis scales. The statistics are calculated only for stations located north from 60°N and with at least 60 years of data during the 1950-2021 period.
